# Supplementary material for: Expression patterns of E2Fs identify tumor microenvironment features in human gastric cancer
Source: PeerJ. 2024 Feb 13;12:e16911. doi: 10.7717/peerj.16911 (PMC10870925; doi:10.7717/peerj.16911)
Supplement: Supplemental Information 2 [file peerj-12-16911-s002.docx]

**Supplementary Table1** Primer of E2F2 and E2F8

| E2F2 primer | |
| --- | --- |
| Forward Primer | CGTCCCTGAGTTCCCAACC |
| Reverse Primer | GCGAAGTGTCATACCGAGTCTT |
| E2F8 primer | |
| Forward Primer | CCTGAGATCCGCAACAGAGAT |
| Reverse Primer | AGATGTCATTATTCACAGCAGGG |
| GAPDH primer | |
| Forward Primer | CTGGGCTACACTGAGCACC |
| Reverse Primer | AAGTGGTCGTTGAGGGCAATG |
