# Supplementary material for: Expression patterns of E2Fs identify tumor microenvironment features in human gastric cancer
Source: PeerJ. 2024 Feb 13;12:e16911. doi: 10.7717/peerj.16911 (PMC10870925; doi:10.7717/peerj.16911)
Supplement: Supplemental Information 3 [file peerj-12-16911-s003.docx]

**Supplementary Table2** siRNA of E2F2 and E2F8

| siE2F2#1 | |
| --- | --- |
| sense（5'-3'） | GCCUAUGUGACUUACCAGGAUTT |
| antisense（5'-3'） | AUCCUGGUAAGUCACAUAGGCTT |
| siE2F2#2 | |
| sense（5'-3'） | CCGAGGGCCAAGUUGUGCGAUTT |
| antisense（5'-3'） | AUCGCACAACUUGGCCCUCGGTT |
| siE2F8#1 | |
| sense（5'-3'） | CAUAAGUUCUUAGCACGAUAUTT |
| antisense（5'-3'） | AUAUCGUGCUAAGAACUUAUGTT |
| siE2F8#2 | |
| sense（5'-3'） | GCCCAGAAAUCAGUCCAAAUATT |
| antisense（5'-3'） | UAUUUGGACUGAUUUCUGGGCTT |
