# Supplementary material for: Expression patterns of E2Fs identify tumor microenvironment features in human gastric cancer
Source: PeerJ. 2024 Feb 13;12:e16911. doi: 10.7717/peerj.16911 (PMC10870925; doi:10.7717/peerj.16911)
Supplement: Supplemental Information 4 [file peerj-12-16911-s004.docx]

**Supplementary Table4** Sequence of overexpression plasmid for E2F2 and E2F8

| Overexpressed E2F2 plasmid |
| --- |
| gcgatcgccaccATGCTGCAAGGGCCCCGGGCCTTGGCTTCGGCCGCTGGGCAGACCCCGAAGGTGGTGCCCGCGATGAGCCCCACAGAGCTGTGGCCATCCGGCCTCAGCAGCCCCCAGCTCTGCCCAGCTACTGCTACCTACTACACACCGCTGTACCCGCAGACGGCGCCTCCCGCAGCGGCGCCAGGCACCTGCCTCGACGCCACTCCCCACGGACCCGAGGGCCAAGTTGTGCGATGCCTGCCGGCAGGCCGGCTGCCGGCCAAAAGGAAGCTGGATCTGGAGGGGATTGGGAGGCCCGTCGTCCCTGAGTTCCCAACCCCCAAGGGGAAGTGCATCAGAGTGGATGGCCTCCCCAGCCCCAAAACCCCCAAATCCCCCGGGGAGAAGACTCGGTATGACACTTCGCTGGGGCTGCTCACCAAGAAGTTCATTTACCTCCTGAGCGAGTCAGAGGATGGGGTCCTGGACCTGAACTGGGCCGCTGAGGTGCTGGACGTGCAGAAGCGGCGCATCTATGACATCACCAACGTGCTGGAAGGCATCCAGCTCATCCGCAAGAAGGCCAAGAACAACATCCAGTGGGTAGGCAGGGGAATGTTTGAAGACCCCACCAGACCTGGGAAGCAGCAACAGCTGGGGCAGGAGCTGAAGGAGCTGATGAACACGGAGCAGGCCTTGGACCAGCTCATCCAGAGCTGCTCTCTGAGCTTCAAGCACCTGACTGAGGACAAGGCCAACAAGAGGCTGGCCTATGTGACTTACCAGGATATCCGTGCTGTTGGCAACTTTAAGGAGCAGACAGTGATTGCCGTCAAGGCCCCTCCGCAGACGAGACTGGAAGTGCCCGACAGGACTGAGGACAACCTGCAGATATATCTCAAGAGCACCCAAGGGCCCATCGAAGTCTACCTGTGCCCAGAGGAGGTGCAGGAGCCGGACAGTCCTTCCGAGGAGCCTCTCCCCTCTACCTCCACCCTCTGCCCCAGCCCTGACTCTGCCCAGCCCAGCAGCAGCACCGACCCTAGCATCATGGAGCCCACAGCATCCTCAGTGCCAGCACCAGCGCCAACCCCCCAGCAGGCCCCACCGCCTCCATCCCTGGTCCCCTTGGAGGCTACTGACAGCCTGCTGGAGCTGCCGCACCCACTCCTGCAGCAGACTGAGGACCAGTTCCTGTCCCCGACCCTGGCGTGCAGCTCCCCTCTGATCAGCTTCTCCCCATCCTTGGACCAGGACGACTACCTGTGGGGCTTGGAGGCGGGTGAGGGCATCAGCGATCTCTTCGACTCCTACGACCTTGGGGACCTGTTGATTAATacgcgt |
| Overexpressed E2F8 plasmid |
| GCGATCGCCATGGAGAACGAAAAGGAAAATCTCTTTTGTGAGCCACATAAAAGGGGACTAATGAAAACACCTCTGAAAGAATCCACCACAGCAAATATCGTGTTGGCAGAGATCCAGCCTGACTTTGGCCCTTTAACCACACCTACCAAGCCCAAGGAAGGCTCTCAGGGAGAGCCGTGGACACCGACAGCCAACCTGAAAATGCTCATCAGTGCTGTG  AGCCCTGAGATCCGCAACAGAGATCAGAAAAGGGGTTTGTTTGACAACAGAAGTGGATTACCTGAGGCCAAAGACTGTATACACGAACACTTATCTGGAGATGAATTTGAGAAATCCCAACCAAGTCGAAAAGAGAAAAGTTTAGGATTATTGTGTCATAAGTTCTTAGCACGATATCCTAATTATCCCAACCCTGCTGTGAATAATGACATCTGCCTTGACGAAGTGGCAGAGGAACTTAATGTTGAACGTCGACGCATTTACGATATCGTGAACGTCC  TAGAGAGTTTACATATGGTGAGCCGCCTCGCCAAAAACAGGTACACTTGGCACGGGCGACACAATCTCAACAAAACCCTTGGCACCTTGAAGAGCATCGGGGAGGAGAATAAGTACGCCGAGCAGATTATGATGATCAAAAAGAAAGAATATGAGCAAGAGTTTGACTTTATTAAGAGTTACAGTATAGAGGATCATATCATCAAATCAAACACTGGCCCAAATGGACACCCAGACATGTGTTTTGTGGAACTCCCTGGAGTGGAATTTCGGGCAGCTTCTGTAAACAGCCGCAAAGACAAGTCTTTAAGGGTAATGAGCCAGAAATTTGTGATGCTGTTTTTGGTGTCAACGCCTCAGATAGTAAGCCTAGAAGTTGCTGCCAAGATTTTAATTGGGGAGGACCATGTGGAAGATTTGGATAAAAGCAAGTTTAAAACAAAAATTAGGAGGTTGTATGATATAGCTAATGTTCTGAGTAGCCTGGATCTTATCAAGAAAGTTCATGTTACAGAGGAAAGAGGCCGAAAACCAGCTTTCAAATGGACCGGCCCAGAAATCAGTCCAAATACCAGTGGCTCCAGCCCAGTCATTCATTTTACTCCCTCTGATTTGGAGGTGAGACGGTCTTCAAAAGAGAACTGTGCCAAAAACCTCTTTTCCACACGTGGGAAACCAAACTTTACTCGACACCCATCTCTTATCAAATTGGTAAAGAGTATAGAAAGTGATCGGAGAAAGATAAATTCTGCGCCCAGTAGCCCTATCAAGACCAACAAAGCTGAGAGTTCTCAGAATTCTGCACCCTTCCCAAGTAAAATGGCTCAGCTCGCAGCTATTTGTAAAATGCAGTTAGAAGAGCAATCAAGTGAATCCAGACAGAAAGTGAAAGTACAGCTGGCAAGATCTGGACCCTGCAAACCAGTAGCCCCTCTGGACCCCCCAGTGAATGCTGAGATGGAGCTGACAGCACCGTCCCTCATCCAGCCCCTGGGAATGGTTCCCCTGATCCCCAGCCCCTTGTCATCAGCAGTGCCCCTGATCCTACCTCAGGCCCCTTCAGGCCCATCCTATGCCATCTACCTGCAGCCCACTCAAGCCCACCAAAGTGTGACGCCACCCCAAGGCCTGAGCCCAACGGTGTGCACCACCCACTCTTCTAAAGCTACTGGCTCAAAAGACTCCACAGATGCCACCACTGAGAAGGCAGCCAATGATACCTCAAAGGCCAGTGCCTCTACCAGGCCTGGAAGCTTGCTGCCAGCACCAGAGAGGCAAGGGGCAAAGAGCCGAACCAGGGAGCCAGCTGGAGAAAGAGGCTCAAAGAGGGCAAGCATGCTCGAGGACAGTGGTTCCAAAAAGAAATTTAAAGAGGACCTAAAAGGACTTGAAAATGTCTCCGCAACCTTGTTCCCATCAGGATACCTAATCCCTCTCACGCAGTGCTCATCCCTGGGGGCAGAGTCCATTTTGTCTGGTAAAGAAAACTCAAGTGCTCTTTCCCCAAACCACAGGATTTACAGCTCCCCAATTGCAGGTGTTATTCCAGTGACATCATCTGAACTCACTGCTGTTAATTTTCCCTCTTTTCATGTAACACCGTTGAAGCTAATGGTCTCACCAACTTCCGTGGCAGCCGTACCTGTCGGGAACAGCCCGGCTCTCGCTTCAAGCCACCCTGTTCCCATCCAGAACCCAAGCTCAGCCATTGTAAACTTCACCCTGCAGCACCTGGGACTCATCTCACCCAATGTGCAGTTGTCTGCCAGCCCTGGGTCTGGAATCGTTCCTGTGTCTCCAAGAATAGAGTCTGTTAATGTCGCACCAGAAAATGCAGGCACTCAGCAAGGAAGGGCCACCAACTATGACTCACCAGTCCCAGGCCAGAGCCAGCCAAATGGACAATCAGTTGCTGTGACAGGGGCACAACAGCCTGTTCCTGTGACACCCAAAGGGTCACAATTAGTGGCCGAAAGTTTCTTCCGTACCCCAGGTGGACCCACCAAGCCAACCAGCTCATCCTGCATGGATTTTGAGGGTGCTAATAAAACCTCCTTAGGAACTCTCTTTGTCCCACAGCGAAAACTGGAAGTCTCAACAGAGGATGTCCATACGCGT |
