# Supplementary figures and images for: Expression patterns of E2Fs identify tumor microenvironment features in human gastric cancer
Source: PeerJ. 2024 Feb 13;12:e16911. doi: 10.7717/peerj.16911 (PMC10870925; doi:10.7717/peerj.16911)

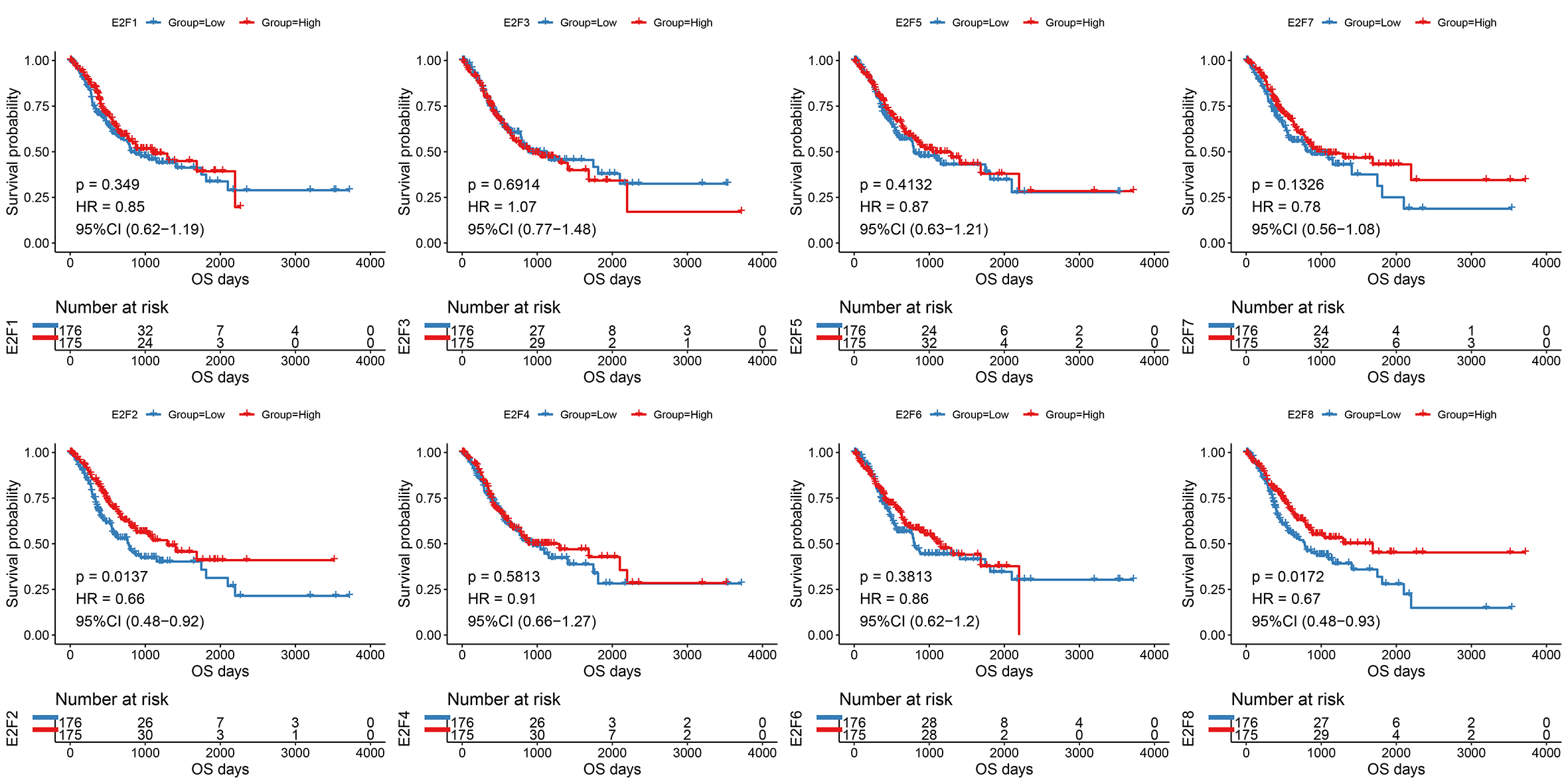

Supplement: Supplemental Information 5 [file peerj-12-16911-s005.jpg]

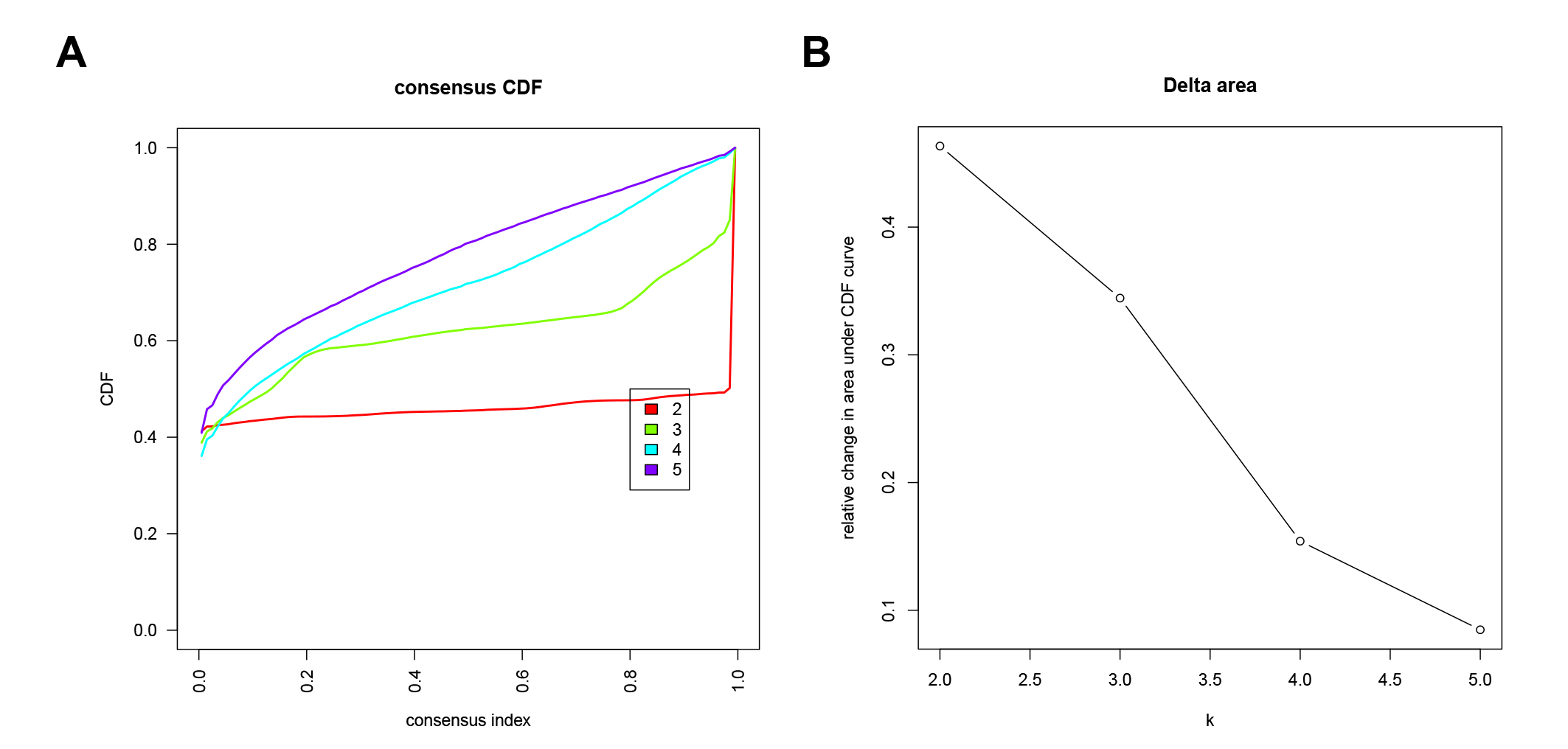

Supplement: Supplemental Information 6 — (A–B) cumulative distribution functions (CDF) of E2F Clusters. [file peerj-12-16911-s006.jpg]

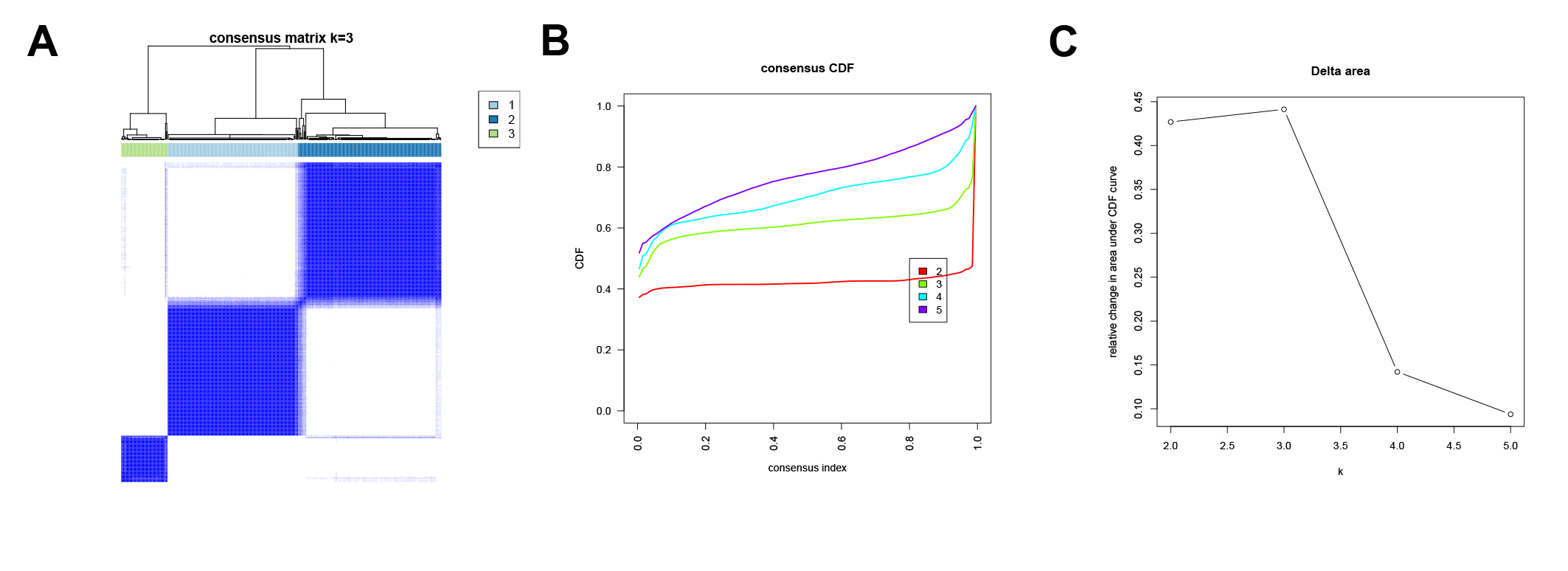

Supplement: Supplemental Information 7 — (A) Consensus clustering matrix for k = 3. (B–C) CDF of E2F gene clusters. [file peerj-12-16911-s007.jpg]

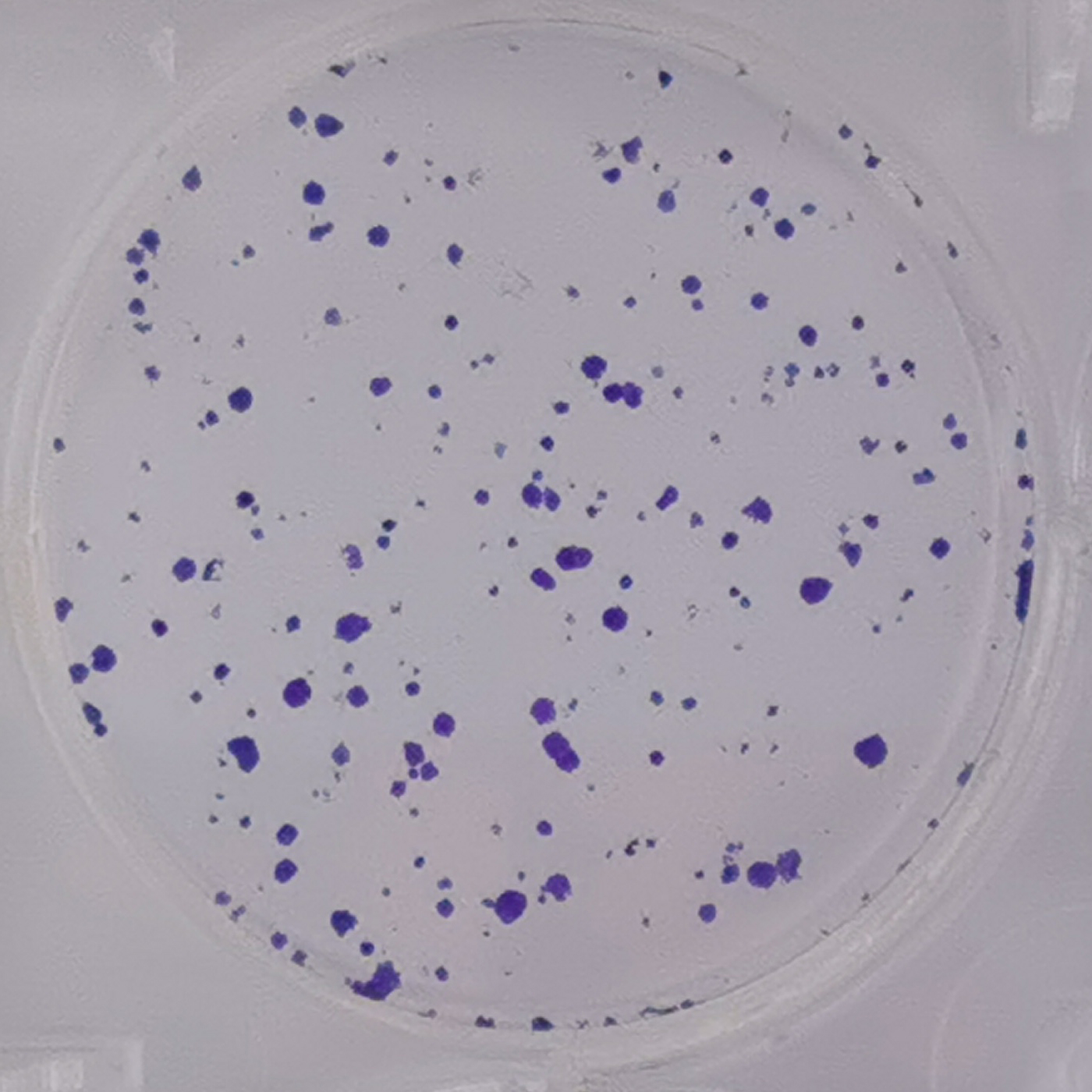

Supplement: Supplemental Information 8 [file peerj-12-16911-s008.zip › Figure8D raw figure/NC.jpg]

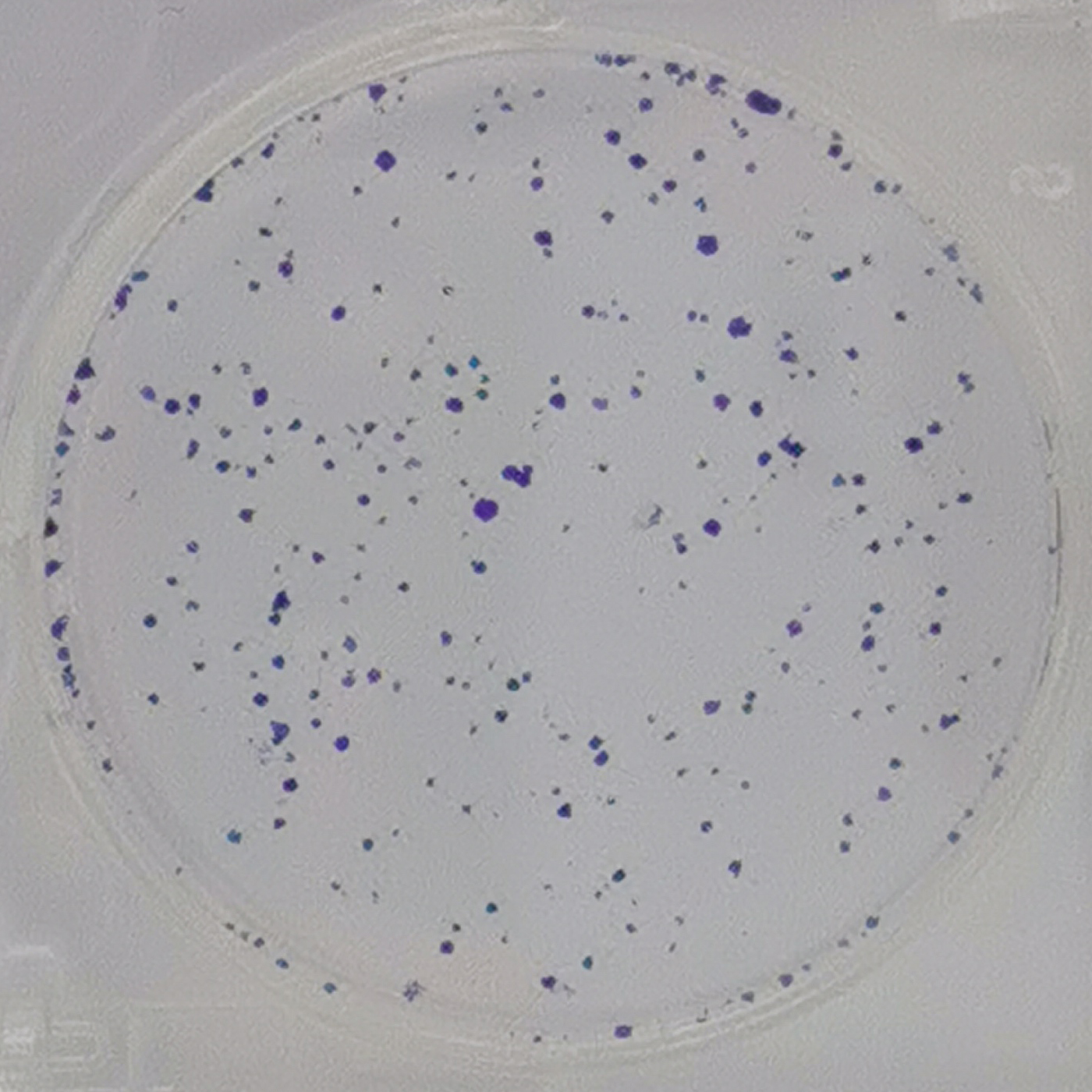

Supplement: Supplemental Information 8 [file peerj-12-16911-s008.zip › Figure8D raw figure/siE2F2#1.jpg]

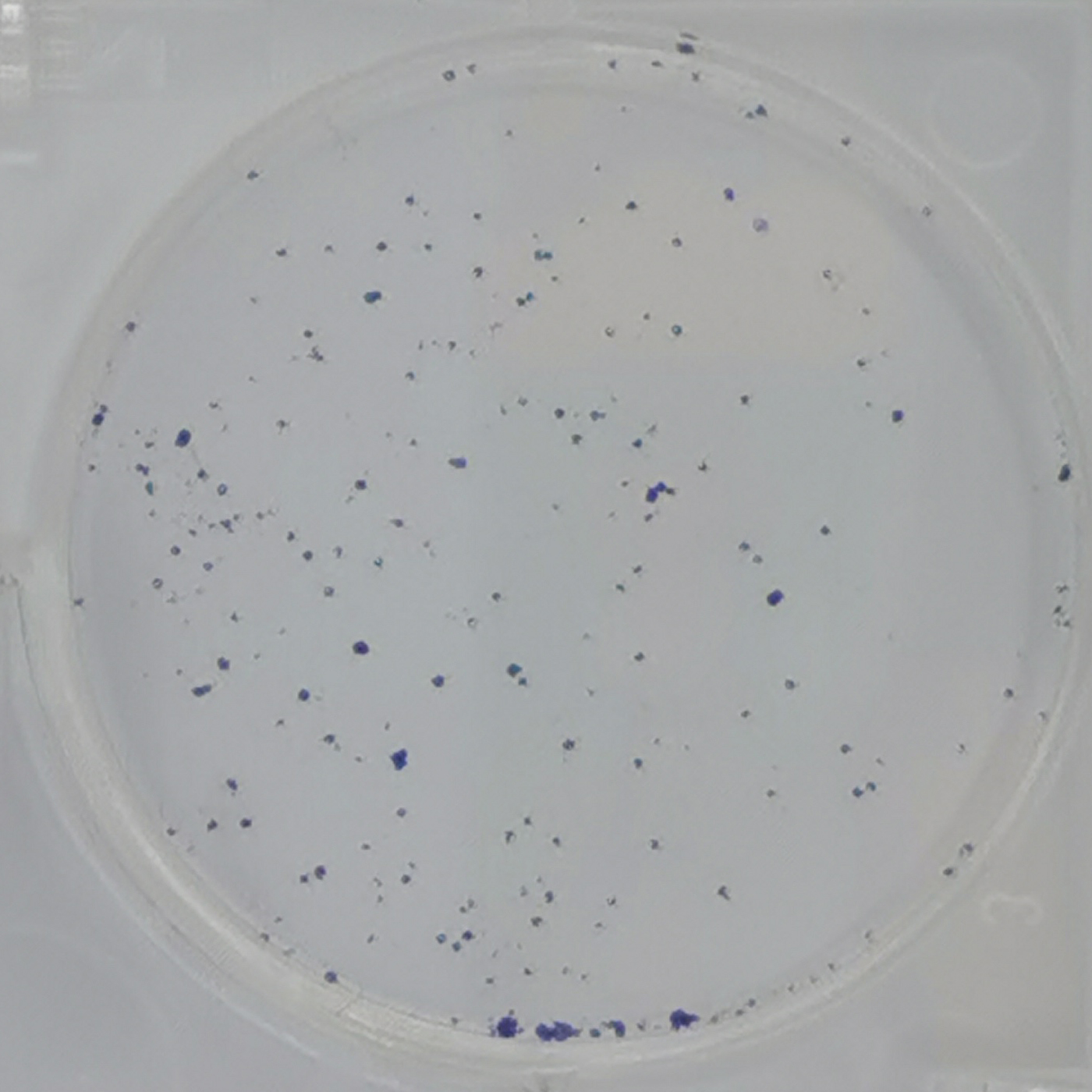

Supplement: Supplemental Information 8 [file peerj-12-16911-s008.zip › Figure8D raw figure/siE2F2#2.jpg]

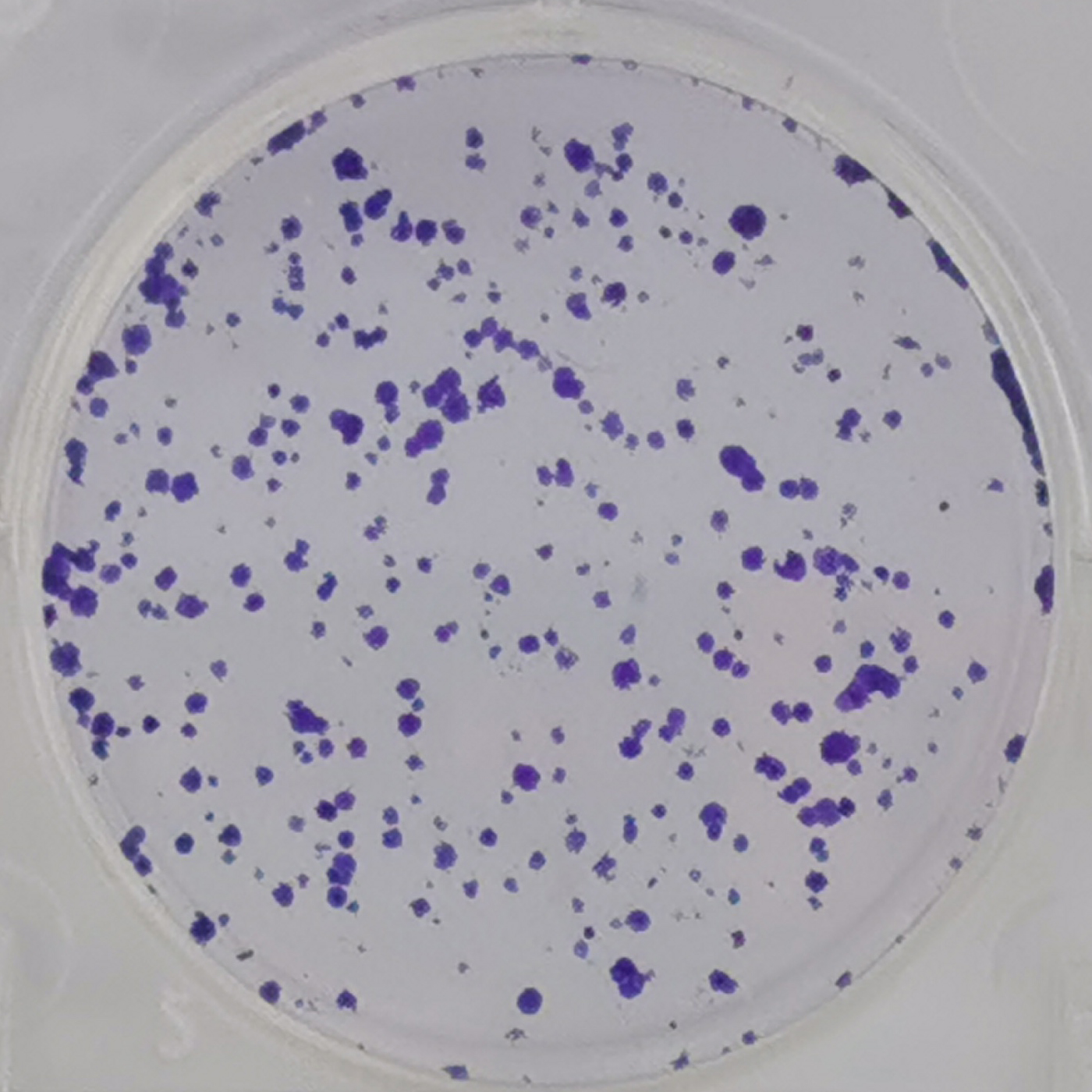

Supplement: Supplemental Information 8 [file peerj-12-16911-s008.zip › Figure8D raw figure/siE2F8#1.jpg]

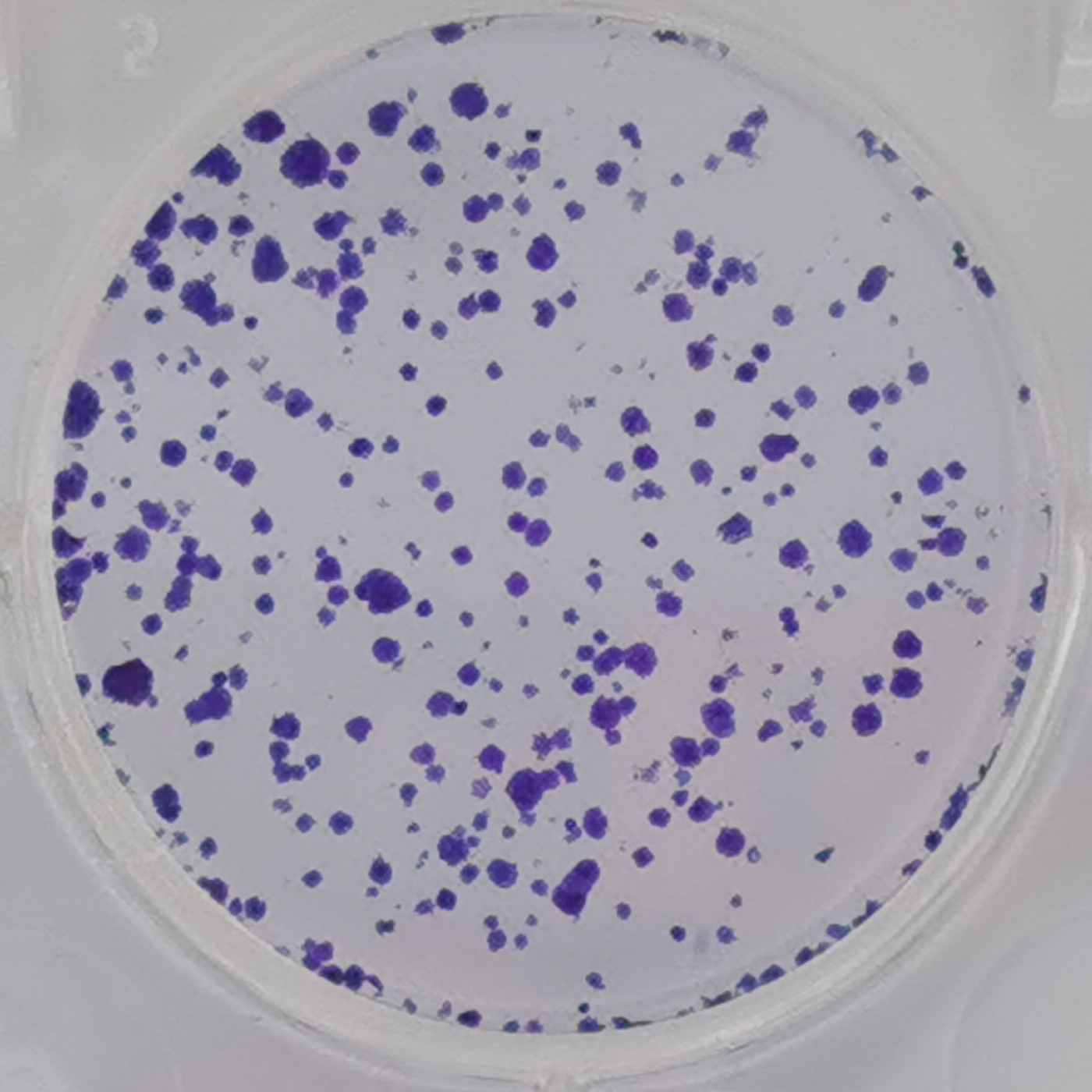

Supplement: Supplemental Information 8 [file peerj-12-16911-s008.zip › Figure8D raw figure/siE2F8#2.jpg]

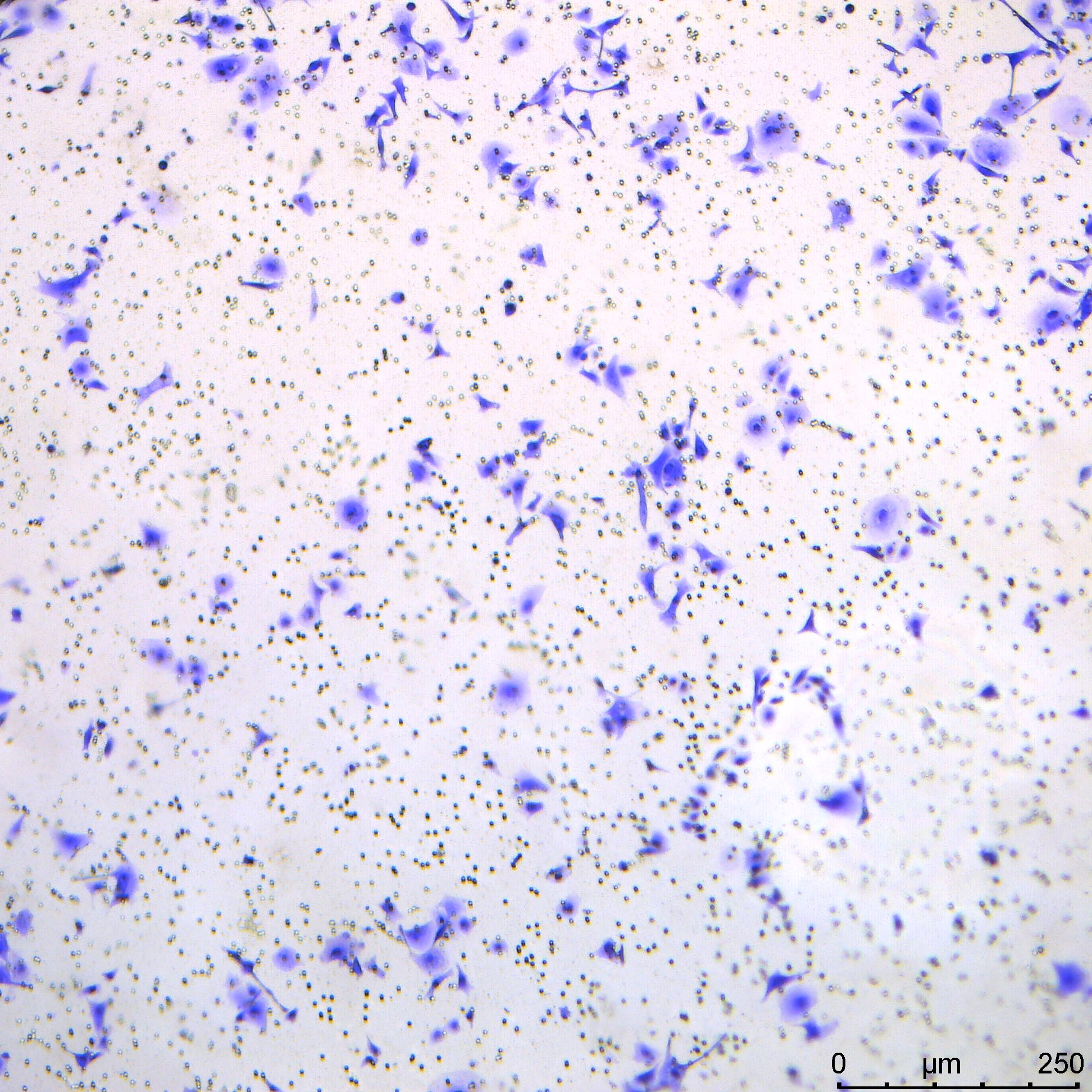

Supplement: Supplemental Information 8 [file peerj-12-16911-s008.zip › Figure8E raw figure/NC.jpg]

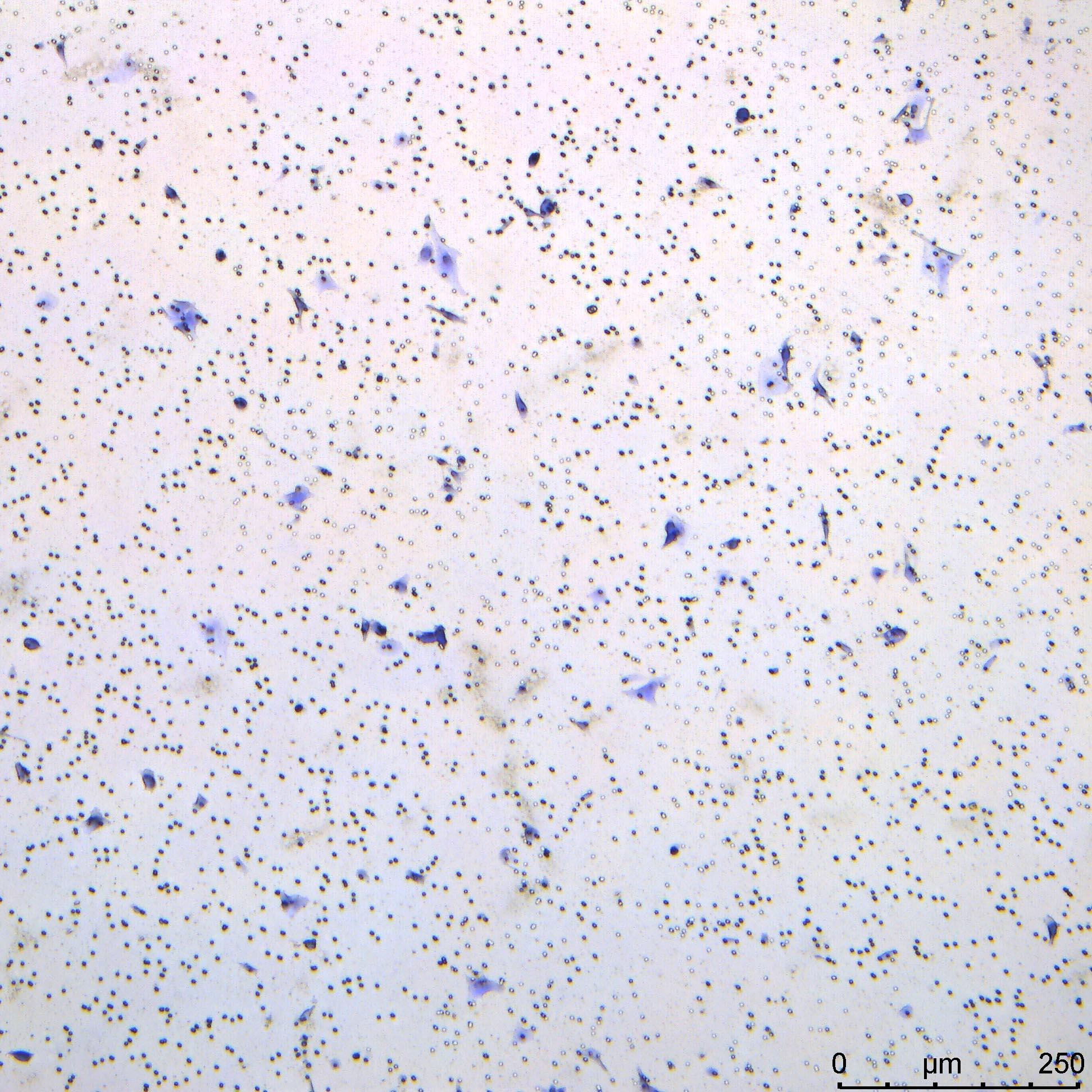

Supplement: Supplemental Information 8 [file peerj-12-16911-s008.zip › Figure8E raw figure/siE2F2-1.jpg]

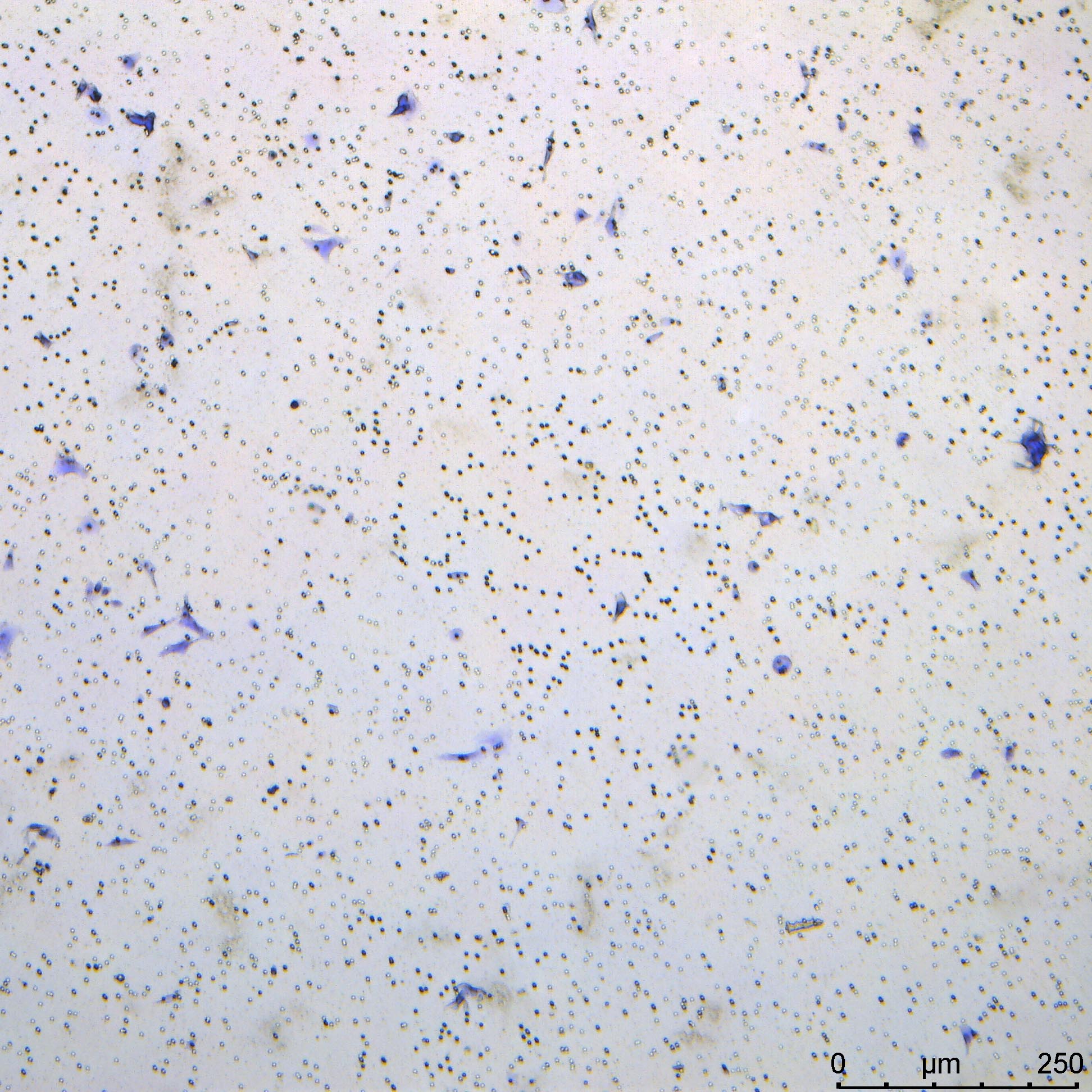

Supplement: Supplemental Information 8 [file peerj-12-16911-s008.zip › Figure8E raw figure/siE2F2-2.jpg]

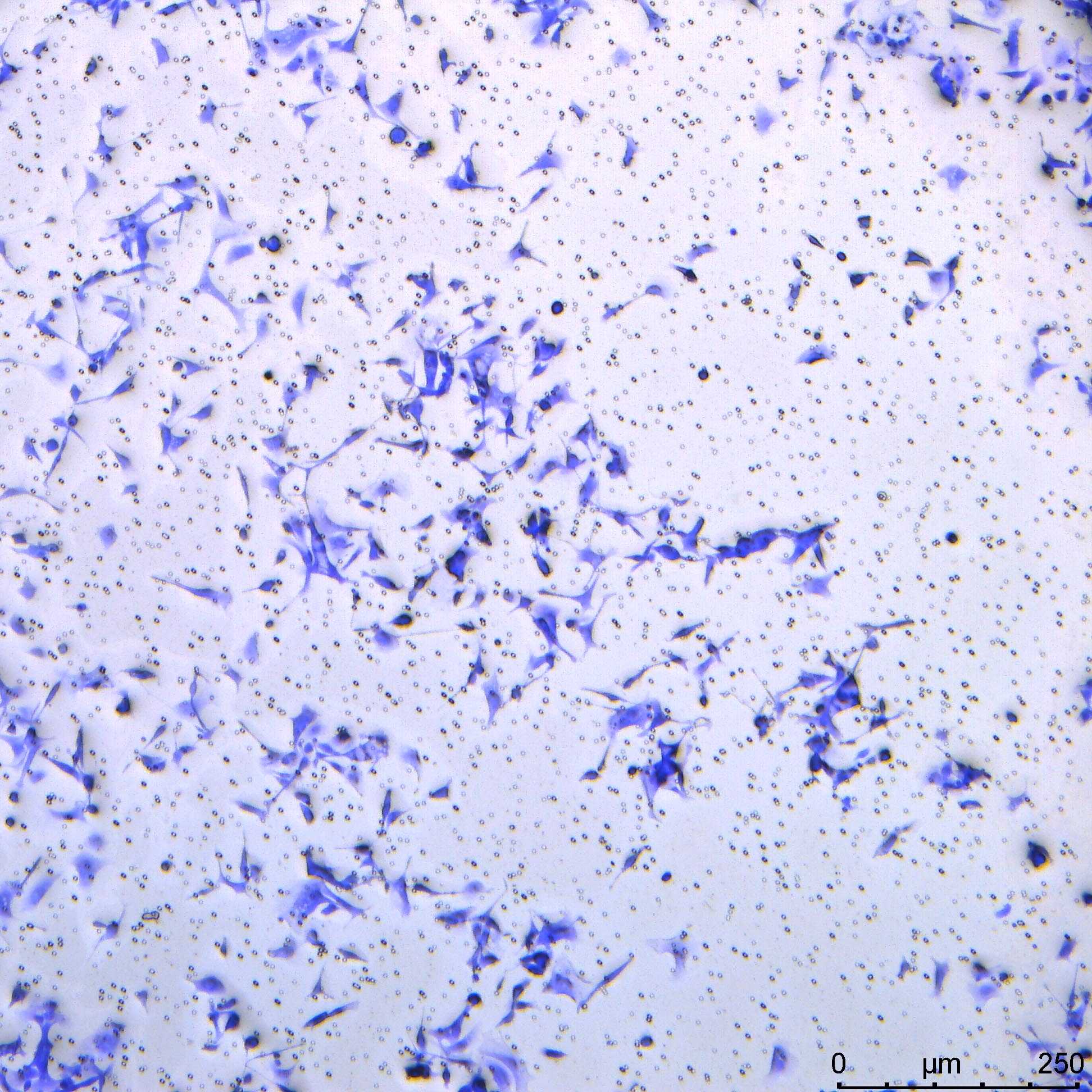

Supplement: Supplemental Information 8 [file peerj-12-16911-s008.zip › Figure8E raw figure/siE2F8-1.jpg]

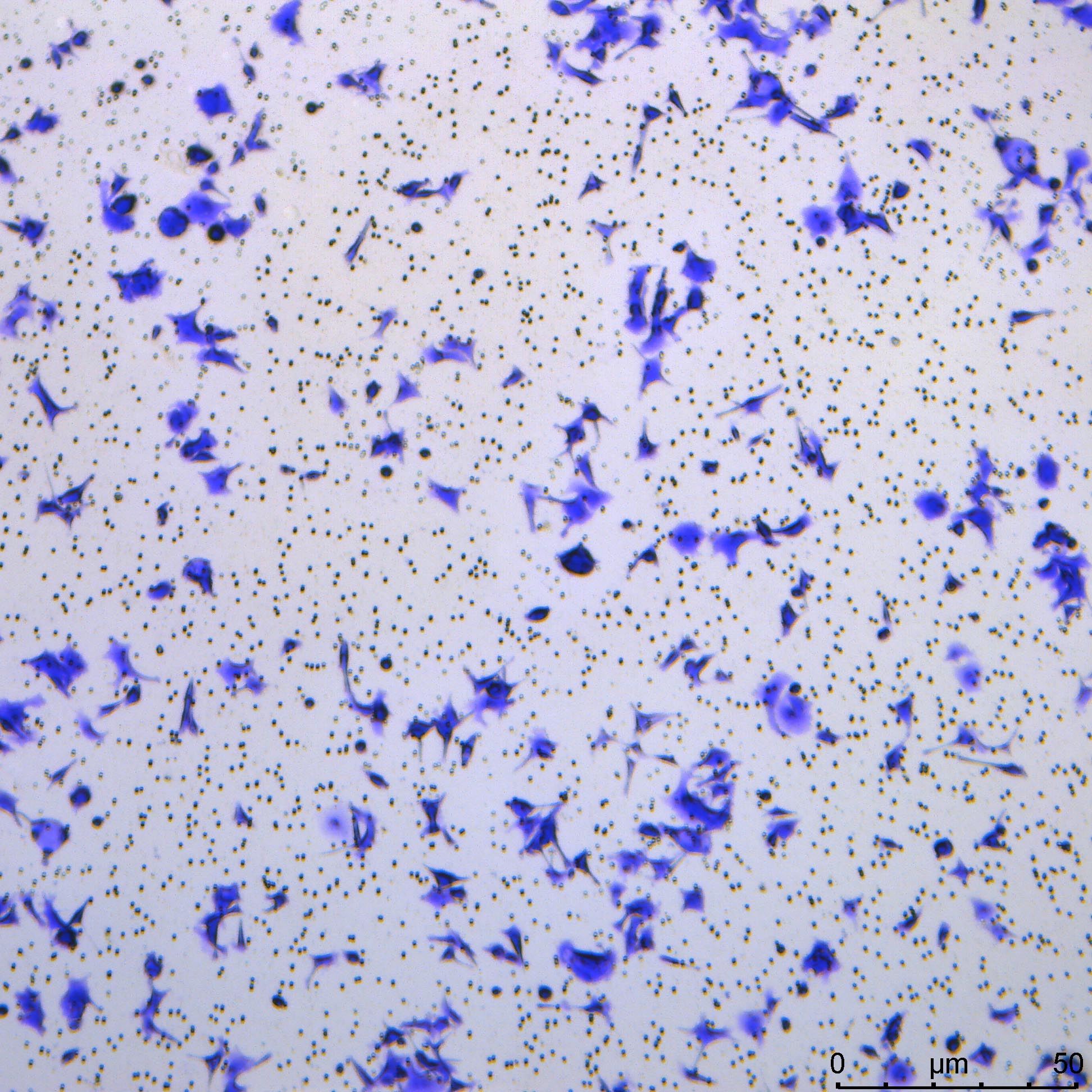

Supplement: Supplemental Information 8 [file peerj-12-16911-s008.zip › Figure8E raw figure/siE2F8-2.jpg]

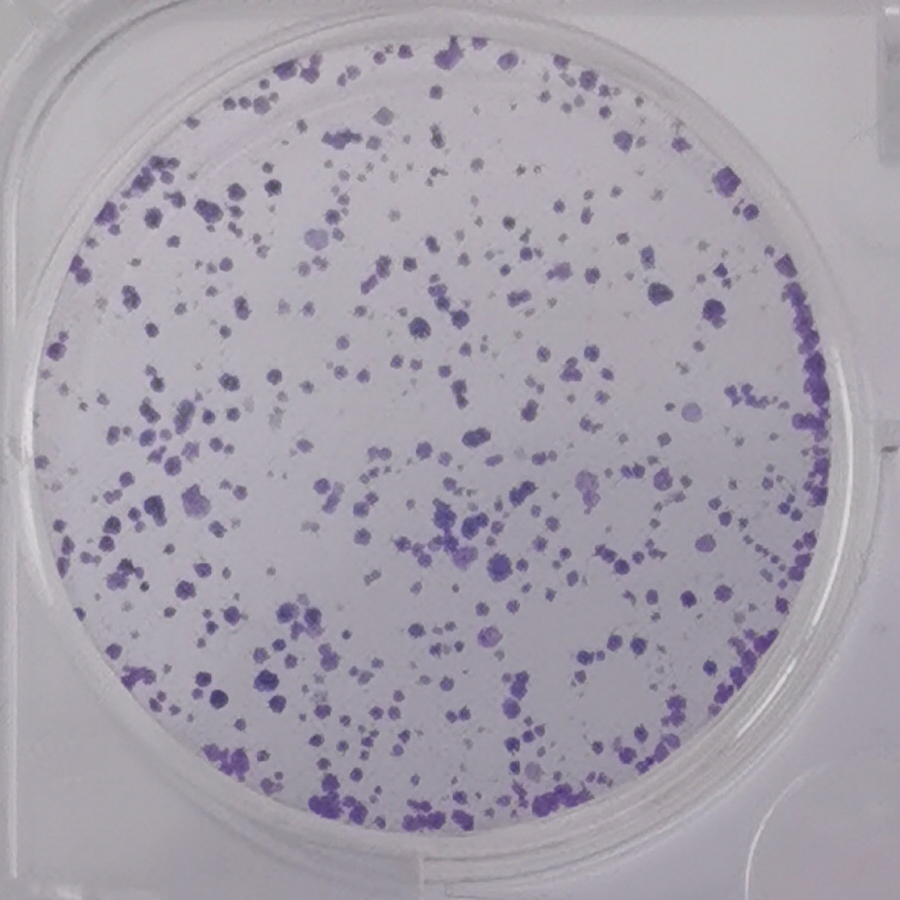

Supplement: Supplemental Information 8 [file peerj-12-16911-s008.zip › Figure8H raw figure/NC.jpg]

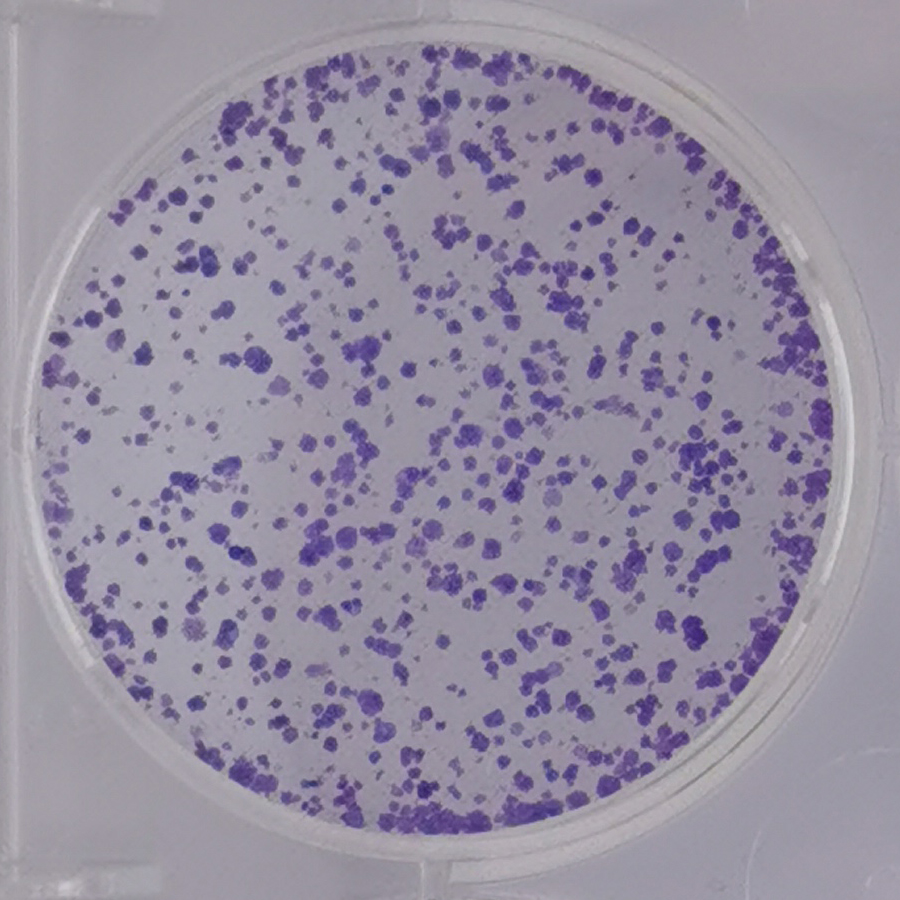

Supplement: Supplemental Information 8 [file peerj-12-16911-s008.zip › Figure8H raw figure/OE-E2F2.jpg]

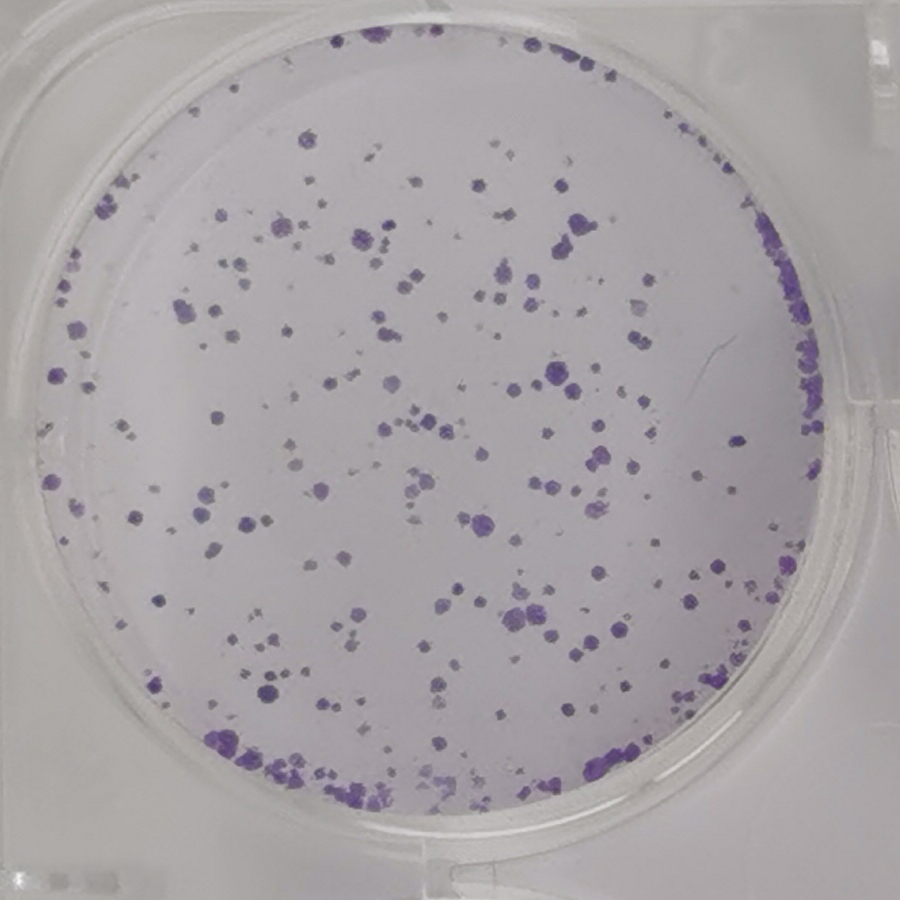

Supplement: Supplemental Information 8 [file peerj-12-16911-s008.zip › Figure8H raw figure/OE-E2F8.jpg]

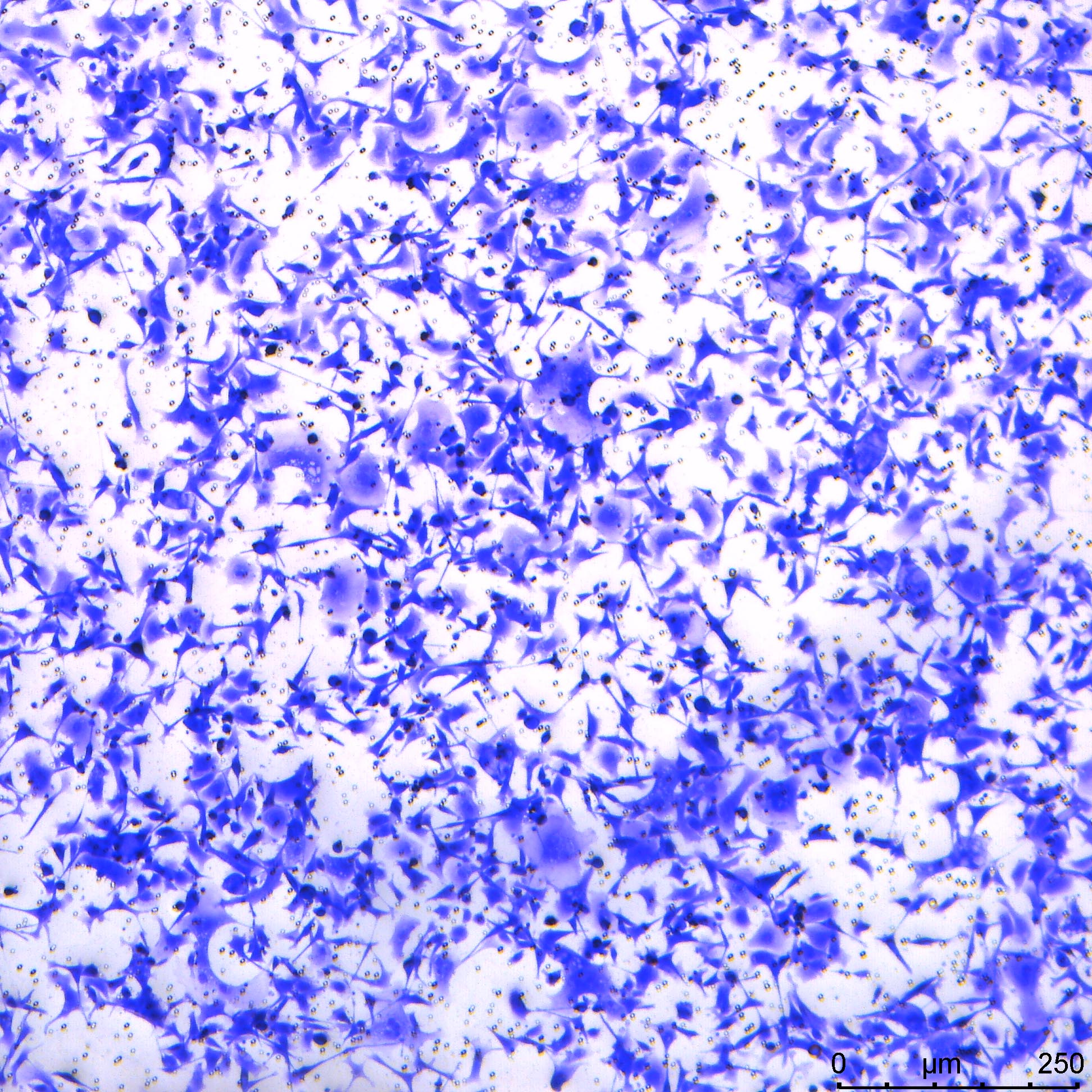

Supplement: Supplemental Information 8 [file peerj-12-16911-s008.zip › Figure8I raw figure/OE-E2F2.jpg]

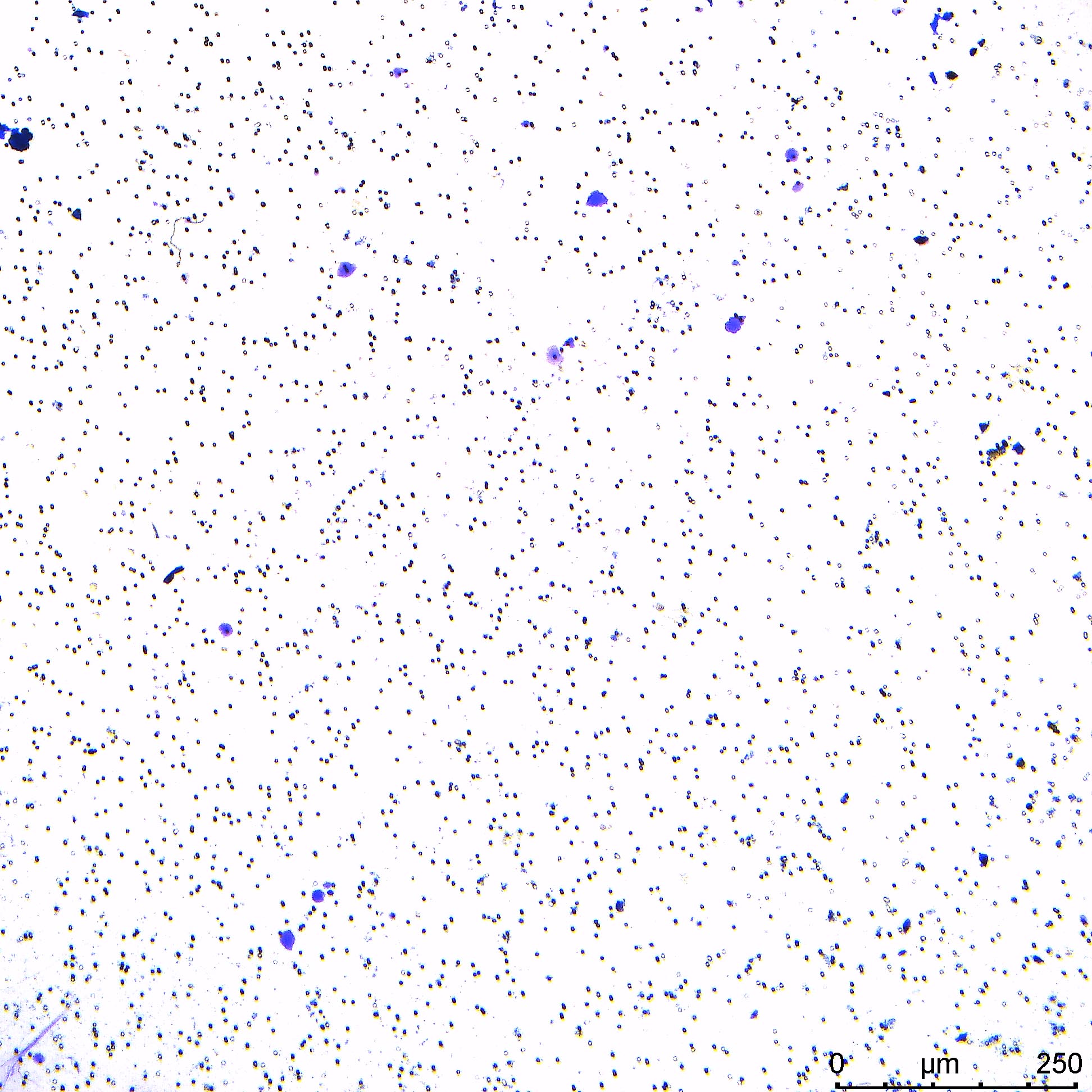

Supplement: Supplemental Information 8 [file peerj-12-16911-s008.zip › Figure8I raw figure/OE-E2F8.jpg]

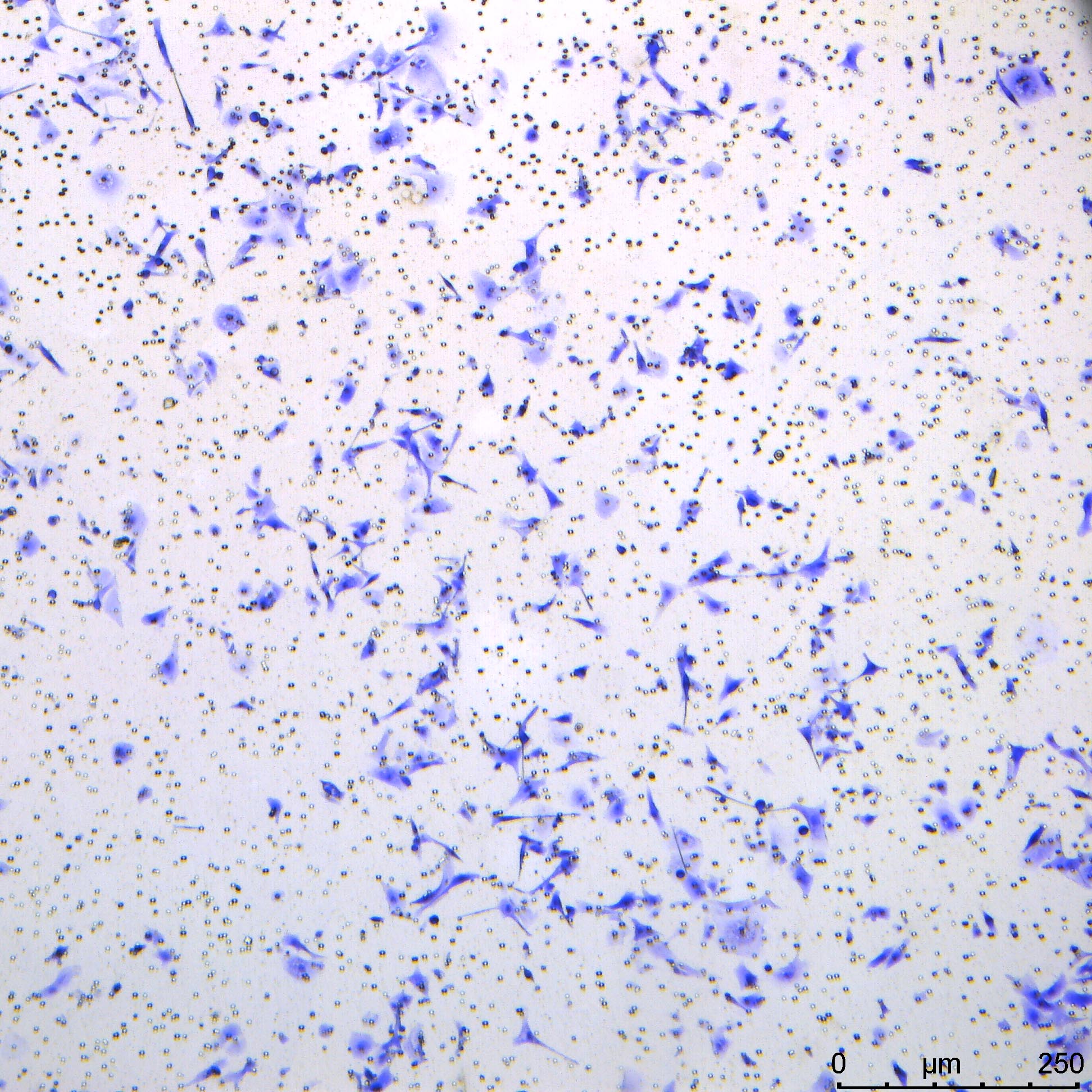

Supplement: Supplemental Information 8 [file peerj-12-16911-s008.zip › Figure8I raw figure/OE-NC.jpg]
